# A Review of Psychophysiological Measures to Assess Cognitive States in Real-World Driving

**DOI:** 10.3389/fnhum.2019.00057

**Published:** 2019-03-19

**Authors:** Monika Lohani, Brennan R. Payne, David L. Strayer

**Affiliations:** ^1^Department of Educational Psychology, University of Utah, Salt Lake City, UT, United States; ^2^Department of Psychology, University of Utah, Salt Lake City, UT, United States

**Keywords:** psychophysiology, cognition, driving, traffic safety, real-world

## Abstract

As driving functions become increasingly automated, motorists run the risk of becoming cognitively removed from the driving process. Psychophysiological measures may provide added value not captured through behavioral or self-report measures alone. This paper provides a selective review of the psychophysiological measures that can be utilized to assess cognitive states in real-world driving environments. First, the importance of psychophysiological measures within the context of traffic safety is discussed. Next, the most commonly used physiology-based indices of cognitive states are considered as potential candidates relevant for driving research. These include: electroencephalography and event-related potentials, optical imaging, heart rate and heart rate variability, blood pressure, skin conductance, electromyography, thermal imaging, and pupillometry. For each of these measures, an overview is provided, followed by a discussion of the methods for measuring it in a driving context. Drawing from recent empirical driving and psychophysiology research, the relative strengths and limitations of each measure are discussed to highlight each measures' unique value. Challenges and recommendations for valid and reliable quantification from lab to (less predictable) real-world driving settings are considered. Finally, we discuss measures that may be better candidates for a near real-time assessment of motorists' cognitive states that can be utilized in applied settings outside the lab. This review synthesizes the literature on in-vehicle psychophysiological measures to advance the development of effective human-machine driving interfaces and driver support systems.

## The Importance of Psychophysiological Measures in Traffic Safety

Suboptimal level of cognitive functioning (e.g., inattention, drowsiness) is a key cause of traffic accidents and poor driving performance. According to Traffic Safety Culture Index, 87.5% of drivers identify distracted driving to be a greater concern today than in past years and 87.9% perceive drowsiness as a threat to their safety (AAA Foundation for Traffic Safety, 2018). Traffic safety researchers are constantly working on methods to improve driving performance by assessing cognitive states, such as drivers' workload, inattention, and fatigue. One way to improve the assessment of covert cognitive states is to adopt a multi-method approach to measure changes in central and peripheral nervous system functioning in order to sense near-real time information about cognitive states of motorists. Such assessments of internal states can also promote the development of Advanced Driver Assistance Systems (ADAS) that can predict and augment risky driving behavior.

### Why Adopt Psychophysiological Measures?

Cognitive states can be assessed using subjective, behavioral, and physiological measures (Mauss and Robinson, 2009; Strayer et al., 2015; Lohani et al., 2018). Subjective measures can be limiting if the assessment is disruptive to the real-time task (i.e., primary task intrusion, see O'Donnell and Eggemeier, 1986). More importantly, humans may not always be accurate in making judgements about their cognitive states (Schmidt et al., 2009). Motorists can be inaccurate in making judgments about their internal and cognitive states (such as their attention, workload, and drowsiness levels). For instance, motorists were inaccurate at self-assessments of vigilance (Schmidt et al., 2009); even though objective physiological indicators (e.g., heart rate, EEG, and ERPs) suggested poor vigilance levels at the end of a 3-h drive, participants self-reported improved vigilance instead (Schmidt et al., 2009). Such misjudgments in assessment of cognitive states suggest that objective measures are required to assess and augment human behavior in order to reduce risk for traffic safety. While behavioral measures (such as head movement detection to assess distraction) are also useful, given the intent of this review, we will focus on physiological measures. Accuracy in detecting cognitive workload has been found to significantly increase when physiological data was utilized (Lenneman and Backs, 2009, 2010; Solovey et al., 2014; Borghini et al., 2015; Yang et al., 2016). Some work has also found that physiological measures were sensitive to variations in cognitive load during secondary tasks while behavioral driving measures like steering wheel reversals and velocity (Belyusar et al., 2015) and lane-keeping measures (Lenneman and Backs, 2009) were not. Unlike behavioral measures (e.g., verbal and facial behavior), many physiological measures are not under voluntary control of motorists. Moreover, cognitive states such as mental workload are a multi-faceted and dynamic concept and self-report alone cannot be used to operationalize it, but multiple measures (e.g., performance and physiology) are warranted (de Waard and Lewis-Evans, 2014). Thus, inclusion of physiological data can complement and extend behavioral metrics and improve assessments of motorists' state-level changes in cognition (Brookhuis and de Waard, 1993; Mehler et al., 2012).

As automation is likely to become more prevalent over time, real-time monitoring behaviors required by motorists may decline as they are less involved in the driving process. This is a critical reason why non-behavior-based metrics will become more relevant to incorporate into our understanding of the motorists' cognitive states. Moreover, distracted motorists of a self-driving vehicle compared to manually driving motorists take longer to gain control of the driving task once automation deactivates (Vogelpohl et al., 2018). Intelligent driving assistance systems should be capable of reliably sensing and assessing distraction and drowsiness levels of motorists to be able to augment safe-driving conditions. Building reliable systems to be able to predict decreased levels of vigilance or dangerous levels of fatigue, drowsiness, or workload could help augment them in a timely manner (Balters et al., 2018).

### Cognition in Dynamic Real-World Driving Contexts

In general, psychophysiological measures can be used to assess degree of arousal or activation (Mauss and Robinson, 2009). Importantly, multiple psychological constructs can influence variations in psychophysiological measures. For instance, heart rate, skin conductance, and electrical activity of the brain are sensitive to many psychological constructs experienced by motorists, such as workload, drowsiness, stress, etc. In the past years, important contributions have reviewed the literature on specific cognitive states, such as workload (Borghini et al., 2014; Costa et al., 2017), distraction (Matthews et al., 2019), drowsiness (Sahayadhas et al., 2012; Borghini et al., 2014), and stress (Rastgoo et al., 2018) in driving research. These reviews provide an understanding of physiological outcomes that can explain variations in specific constructs based on carefully manipulated and well-controlled designs. Unlike highly controlled lab-based settings, where a single construct (e.g., workload) can be successfully manipulated and its effect on psychophysiological measures examined, real-world settings are more dynamic and complex.

In a real-world setting, the net resulting cognitive state of a motorist is a combination of variation among several interrelated constructs (e.g., attention allocation, stress, workload, fatigue). Broadly speaking, the net cognitive state of a motorist, composed of variation among these many dimensions, can be classified along an arousal-spectrum ranging from lower-arousal and passive states, to a state of optimal performance, to a hyper-aroused or over-active state. Indeed, this concept is not new; Yerkes and Dodson (1908) established strong non-linear relationships between arousal-level and performance, and such relationships have since been well-established across many human performance domains (Hebb, 1955; Broadhurst, 1959; Wekselblatt and Niell, 2015). Although these ideas are not new, there has been a recent resurgence in a formal understanding of arousal-performance relationships, including an expanded understanding of the underlying neuromodulatory systems involved in regulating task engagement and optimal performance (e.g., the adaptive-gain control theory, Aston-Jones and Cohen, 2005). Given the recent increase in understanding of the mapping between physiological indices of arousal and human performance in the lab, such models serve as a clear starting point in delineating the predictive capacity of psychophysiological measures for understanding cognitive states and human performance in the vehicle.

For instance, low-arousal states relevant to the driving task can be driven by a combination of psychological constructs including low workload, reduced stress, and high drowsiness. On the other hand, an over-aroused state could be due to a combination of high workload and high stress in the presence of low drowsiness. Similarly, other combinations of constructs can also lead to changes in general arousal states as well. Given the likely dynamic interplay among these interrelated constructs in applied settings, the current review focuses on psychophysiological measures that can be utilized to capture motorists' states in real-world driving settings. Indeed, one major applied goal of this work is to be able to accurately capture the dynamic and highly variable changes in arousal that occur in ecologically valid driving settings, a goal that is critical for building accurate predictive models (Yarkoni and Westfall, 2017) of individual motorist's states and future driving performance.

Specifically, there are two novel contributions of this review. First, instead of focusing on a selective construct and related measures of interest, the goal of this current review is to focus on psychophysiological measures that may have the potential to be adopted in real-world and applied settings to measure state level variations in motorists. The paper provides a broad but selective review of a number of psychophysiological measures that we believe show the greatest promise in their utilization to assess low-arousal vs. over-arousal (passive vs. over-active) states in real-world driving environments. The most commonly used physiology-based measures of cognitive states are considered as potential candidates relevant for driving research. The following physiological measures are reviewed (see section “Psychophysiological Measures to Assess Cognitive States” and Tables 1, 2) in assessing arousal state in real-world driving research: electroencephalography and event-related potentials, optical imaging, heart rate, and heart rate variability, blood pressure, skin conductance, electromyography, thermal imaging, and pupillometry. As reviewed in classical contributions by Cacioppo et al. (Cacioppo and Tassinary, 1990; Cacioppo et al., 2007), inference of unique psychological constructs based on physiological indices (one-to-one relation) is still unresolved and is not the aim of this review (see further discussion in section “Research Applicability in Real-World Settings”). However, we discuss how multiple measures (that are sensitive to several interrelated internal states) may be combined to delineate net resulting changes across multiple inter-related cognitive state-level variations. Second, for each measure, we make the distinction between useful research measures and practical measures for real-world application (see section Research Applicability in Real-World Settings and Table 2). Throughout, we have tried to highlight the practical relevance of measures in the driving context. Although this review focuses primarily on on-road and simulated driving contexts, when relevant, we have also drawn research from related contexts (traffic operators, pilots, or ship navigators) to more thoroughly characterize each measure.

**Table 1:** Overview of relationships between arousal state and physiological indices in real-world driving.

**Measure**	**Under-arousal state**	**Over-arousal state**
Electroencephalogram	• Increased alpha due to increase in drowsiness and attentional withdrawal^b^ • Changes in theta and delta activity related to transition to fatigue	• Increase in theta activity due to mental workload • Alpha activity suppression due to workload
Event related potential	• Reduced ERP amplitudes with fatigue, time on task, and lower vigilance over time while driving	• Also, reduced ERP amplitude to driving relevant stimuli under high workload
Optical imaging for cerebral flow	• A decrease in cerebral oxygenation with fatigue and drowsiness	• An increased concentration of oxygenated hemoglobin and a decreased concentration of deoxygenation with mental workload and stress
Heart rate and Heart rate variability^b^	• Decrease in heart rate with drowsiness, decrease in vigilance, use of self-driving technology • Increase in HRV indices (e.g., RMSSD) with drowsiness, fatigue, and disengagement	• Increase in heart rate with mental workload and stress • Decrease in HRV with workload, stress, and vigilance
Blood Pressure^b^	• Decrease in blood pressure relative to baseline with fatigue and drowsiness	• Increase in systolic BP with workload and stress
Electrodermal activity	• Lower EDA relative to baseline activity range with drowsiness	• EDA increase with workload, stress, lower trust in automation, and anxiety
Electromyography	• Decreases in mean and median power frequency of EMG due to decline in muscle activities and fatigue^a^	• High muscle activity relative to baseline with stress
Thermal imaging	• Temperature around baseline levels^a^	• Higher task difficulty increases forehead temperature and decreases nose temperature and thus an increase in the difference between forehead and nose temperatures
Pupillometry	• Decreases in average pupil diameter with drowsiness • Increases in standard deviation of pupil diameter	• Increases in pupil diameter with cognitive load • Decreases in standard deviation of pupil diameter

a *Limited findings available*.

b *Mixed findings reported*.

**Table 2:** Tentative framework for considering the research applicability of these measures in lab and real-world settings.

**Measure**	**Lab-setting**	**Real-world**	**Advantages**	**Disadvantages**
Electroencephalogram	High	High to medium	• High temporal resolution • Direct measure of neural activity	• Contact sensors • Longer setup time
Event related potential	High	Medium^a^	• Same benefits as EEG • Well-characterized components (e.g., P3) • High temporal resolution	• Same disadvantages as EEG • Generally needs higher number of trails and post-processing • Needs time-locking event • Interpretation of amplitude is very context specific
Optical Imaging for Cerebral Flow	High	Low to medium^a^	• Higher spatial resolution • Feasible in naturalistic settings with technical advancements	• Lower temporal resolution (e.g., fNIRS) • Need systematic investigation/replication
Heart Rate/Heart Rate Variability	High	High	• Higher signal-to-noise ratio (SNR) • Easy to collect	• Very sensitive to artifacts • Sensitive to variation in respiration
Blood Pressure	High	Medium^a^	• Reliable • Higher SNR	• Limitations of equipment; can disrupt driving task
Electrodermal activity	High	High	• Sympathetic activity • Easy to collect	• Lagged response • Not all individuals show EDA response
Electromyography	High	Low^a^	• Reliable • High temporal resolution	• Slightly longer setup time • Sensitive to movement • Lower SNR
Thermal Imaging	High	Medium^a^	• Low setup time • Promising technology • Non-contact	• Need systematic investigation/replication
Pupillometry	High	Low^a^	• Non-contact • Quick setup time	• Signal strongly sensitive to variable lighting conditions (pupillary light reflex)

a *Limited findings available*.

## Psychophysiological Measures to Assess Cognitive States

### Electroencephalogram (EEG) and Event-Related Potentials (ERP)

#### EEG Quantification

The *EEG* is a record of both oscillatory and aperiodic brain electrical activity. Neural activity (largely post-synaptic potentials) from multiple simultaneous generators propagate throughout the brain and skull and summate at a distance, where voltages can be measured relatively non-invasively via electrodes placed on the scalp. The dominant sources of scalp-recorded EEG come from cortical pyramidal cells arranged in the columnar organization of the cortex (Nunez and Srinivasan, 2006). Pyramidal cells are the most numerous cortical excitatory cell type and play a critical role in advanced cognitive functions (Spruston, 2008). The laminar organization of the cortex results in cortical pyramidal cells following an *open-field* alignment with a consistent orientation that is perpendicular to the skull, such that their post-synaptic potentials can summate at a distance. Importantly, EEG allows for a high temporal resolution (millisecond) and direct record of neural activity. This detailed temporal resolution also allows for a decomposition of the time-domain EEG signal into spectral information via Fourier analysis, allowing for an examination of oscillatory activity in canonical frequency bands (e.g., alpha, ~8–12 Hz; theta, ~4–7 Hz), which have been related to specific neurocognitive functions. For instance, mental workload increases theta power and reduce alpha power activity (Mun et al., 2017), whereas fatigue increases alpha power (Käthner et al., 2014). Moreover, the development of novel computational techniques for analyzing spectral activity has promoted a wide range of new tools for probing ongoing neural dynamics during human cognition via EEG; such as cross-frequency coupling, phase coupling (Cohen, 2011), independent component analysis (Dasari et al., 2017), and neighborhood component analysis (Lim et al., 2018). In addition, more traditional analyses of transient neural activity that is tied to specific perceptual, motor, or cognitive events can be gleaned from continuous EEG, via the calculation of event-related brain potentials.

#### ERP Quantification

ERPs are electrophysiological responses that are consistently linked in time with specific sensory, cognitive, or motor events. They are derived from the continuously recorded EEG by time-aligning epochs of EEG relative to an event of interest, such as a stimulus onset or a participant's response and averaging many of these similar EEG segments to reveal activity that is time and phase locked to the event. Such discrete events can be added in the experimental design, e.g., every time a participant responded to a secondary task while driving. The logic of this approach is that systematic activity that is locked in time and space to some specific activity will remain in the averaged ERP waveform, whereas activity that is not time- and phase-locked will average to zero with a large enough number of trials (Luck and Kappenman, 2012). The resulting ERP waveform is plotted as voltage over time at a given set of electrodes. ERP topography can also be examined, showing the distribution of activity over the entire space within a particular time-window. A major benefit of ERPs is that the waveform has characteristic *components*, stereotyped features of the ERP with specific eliciting conditions. ERP components are defined empirically by a combination of their polarity, timing, scalp distribution, and sensitivity to task manipulations.

Extensive work has characterized and validated specific ERP components with respect to their associations with specific cognitive and neural processes (e.g., Fabiani et al., 2007; Luck and Kappenman, 2012; Mun et al., 2017). Cognitive demands can modulate several ERP components, such as P3 (discussed below; Käthner et al., 2014), mismatch negativity (MMN is a negative ERP component sensitive to pre-attentive information processing; Wanyan et al., 2018), and late positive potentials amplitude (a later ERP component like P6 that is related to attentional allocation similar to P3; Mun et al., 2017). The P3 component is associated with attentional and memory processes required to detect any changes in incoming stimuli-related information (Polich, 2007). The canonical P3 has two distinct but related components – the P3a and P3b (see Polich, 2007 for a review). The P3a, with an anterior distribution, is associated with novel stimulus-driven attentional processing or orienting responses. The P3b, with a centro-posterior distribution, is associated with task-relevant stimulus-driven attentional, decision making, and subsequent memory processing (Polich, 2007). Both components have been used in driving research. Recent work has also examined how neural indices (as measured by both P3 ERP components) are associated with subjective workload (as measured by NASA-TLX) and how this covariation is influenced by cognitive effort (Yakobi, 2018). Novel techniques (such as intra-block averaging of ERP amplitudes; Horat et al., 2016) can enable robust electrophysiological measurement of cognitive demands over time. Thus, ERPs are an attractive measure for studying cognitive states and performance in driving contexts.

#### EEG/ERP in Driving Context

EEG and ERPs have a long history in the study of the neural indices of cognitive effort and attention allocation in both laboratory and applied settings. EEG is perhaps one of the most widely used neurophysiological methods to study driving behavior. Several frequencies (e.g., power in alpha frequency band) and time (e.g., P3) domain indices can reliably measure changes in cognitive demands (Käthner et al., 2014). This makes EEG is viable measure for applied driving settings.

##### Over-arousal in driving context

Over-aroused states, such as increased workload while driving can be indexed by decreases in alpha power and increases in theta power (Borghini et al., 2014; Käthner et al., 2014). A recent study found alpha band power to be higher during the relaxed condition compared with the engaged condition in an autonomous driving setting (Zander et al., 2017). This highlights the sensitivity of alpha power to internal factors such as attentional engagement. In addition to internal factors, external factors (such as task load and time on task) can also influence alpha and theta power bands in opposite directions (Wascher et al., 2018). For instance, a decrease in task load and time on task led to an increase in relative alpha power, but a decrease in theta power (Getzmann et al., 2018; Wascher et al., 2018). To account for both power bands, past work has also used a ratio of frontal theta and parietal alpha power spectral density to operationalize workload in pilots (Borghini et al., 2015). This ratio approach may be relevant for driving research as well, however this has been a point of debate, as discussed shortly.

The application of known ERP indicators of attentional workload (and their eliciting tasks) can be successfully translated into the driving domain as well. One of the most commonly adopted components in driving research is the P3b (Brookhuis and de Waard, 2010; Solís-Marcos and Kircher, 2018). Mental workload can be indexed by increases in P3b latencies (Ying et al., 2011) and amplitude (Strayer and Drews, 2007). For example, Strayer and Drews (2007) examined the amplitude of the P3b time-locked to the onset of a pace break light under single-task driving conditions or dual-tasking via cell-phone–induced distraction. Drawing on basic experimental work that has shown that the P3b is sensitive to the degree of attention allocated to a task (e.g., Sirevaag et al., 1989), they also showed that cell-phone induced distraction resulted in reduced P3b amplitudes to brake lights. Similar effects have been observed in comparing the workload of “single-task” driving in laboratory simulator vs. real-life driving contexts, where for example, the diversion of attention to other concurrent activities in the vehicle result in additional attentional demands in real-world driving (Strayer et al., 2015).

A recent study compared mental workload due to increased information processing demands consumed by in-vehicle information systems (Solís-Marcos and Kircher, 2018). They found both P3b and N1 latencies and amplitudes to be sensitive to cognitive demands of processing additional in-vehicle information systems. For instance, P3b amplitudes decreased with additional information processing related tasks (Solís-Marcos and Kircher, 2018). P3a amplitude was also found to decrease with additional task-related load (Getzmann et al., 2018). High mental workload has been associated with increased latencies in MMN during driving (Ying et al., 2011) and also increased frontal MMN in flight simulation tasks (Wanyan et al., 2018), however a recent study did not find workload to influence MMN amplitudes (Getzmann et al., 2018). Future work will help clarify sensitivity of MMN in driving research.

##### Under-arousal in driving context

Extensive work has focused on electrophysiological indicators of under-arousal via EEG. A substantial number of papers have implicated changes in alpha amplitude during fatigued driving (e.g., Schier, 2000; Jensen and Mazaheri, 2010; Simon et al., 2011; Zhao et al., 2012; Borghini et al., 2014; Jagannath and Balasubramanian, 2014; Arnau et al., 2017; Brouwer et al., 2017), such that fatigued driving is associated with increased alpha activity. However, other work has challenged these alpha power links with fatigue and claim that alpha power changes may be due to the decreases in task-demands and visual input during monotonous driving tasks and not due to decline in cognitive processing abilities (Wascher et al., 2014). Increases in relative alpha band power with increased time on task, easier driving route, and lower control of driving situations, which suggested that relative alpha power increases imply attentional withdrawal and not fatigue (Wascher et al., 2014, 2018). Wascher et al. (2014, 2018) have argued that mid-frontal theta activity may be a more appropriate neural marker of cognitive-control related processes in driving than occipital alpha activity. Low task load is associated with relatively reduced theta activity, which suggests that theta activity is sensitive to declines in cognitive processing ability. Instead of alpha activity, Wascher et al. (2014, 2018) recommend that indices of oscillatory synchronization (e.g., inter-trial phase clustering) and ERPs (such as P3a) are more reliable and valid indices of changes in cognitive state associated with mental fatigue. For instance, time on task (Wascher et al., 2014), fatigue (Massar et al., 2010), and decreases in vigilance over time (Schmidt et al., 2009) were found to reduce P3a amplitude while driving. Similarly, mind-wandering during driving is associated with a reduction in P3a amplitude (Baldwin et al., 2017). One other study found both P3a and P3b components' amplitudes were reduced due to driving-related fatigue (Guoping and Zhang, 2009). These findings show that ERP components could be utilized to detect variations in neurophysiological arousal due to interrelated cognitive constructs in driving contexts.

Some researchers have argued that LF/HF ratios (e.g., frontal theta/beta) are potential biomarkers for attentional control, and have established some evidence that such measures have good psychometric properties, for e.g., test-retest reliability (Putman et al., 2014; Angelidis et al., 2016). Decreases in beta power (e.g., Zhao et al., 2012; Jagannath and Balasubramanian, 2014) have been found, along with changes in theta and delta activity as markers related to transition to fatigue. This has led some researchers to propose spectral ratio indices (e.g., alpha/beta; Eoh et al., 2005; Wang et al., 2018), as biomarkers of alertness. However, ratio indices have also been criticized for being an inadequate method because it combines frequency bands with distinct topographic specificity that change differently over time (Wascher et al., 2014). There is existing criticism of this ratio approach, especially in driving research (Wascher et al., 2018), and more broadly, researchers in cognitive electrophysiology have been moving away from such highly constrained “band-based” approaches given their lack of replicability across studies. Alternatively, researchers have increasingly endorsed methods that allow for broad-band assessment of spectral dynamics (e.g., 1/f scaling, Voytek and Knight, 2015) and methods that can address narrow-band dynamics without a priori selection of frequency (e.g., cluster-based permutation testing in time-frequency data; Maris and Oostenveld, 2007). Other recent work has used EEG-based detection algorithms to detect fatigue and drowsiness (Li et al., 2017; Morales et al., 2017; Belakhdar et al., 2018; Gao et al., 2018; Wei et al., 2018). However, other work reported no additional benefit of utilizing EEG measures in drowsiness and fatigue detection in sleep deprivation contexts (Perrier et al., 2016; Liang et al., 2017). Another line of work has aimed to apply machine-learning techniques to brain computing interfaces in order to classify states of drowsiness and fatigue in real-time (e.g., Lin et al., 2005; Correa et al., 2014). Recent work has also shown data filtering and processing techniques such as artifact subspace reconstruction and independent component analysis could be utilized for “online” processing of EEG data collected while driving in order to attenuate movement-and noise-related artifacts (Krol et al., 2017). Together, these findings suggest that EEG and ERPs can be utilized as objective techniques to assess state-level variations in cognitive demands.

#### Practical Considerations

There are a number of important considerations when applying EEG indices to real-world driving environments. Typical EEG artifacts arising from muscle-and-eye movements (de Waard, 1996; Zander et al., 2017), impedance shifts, environmental line (60 Hz) noise, and other complications are potentially amplified in real-world environments. As such, real-time monitoring of good quality EEG signals is critical for effective data collection. The commercial introduction of high-impedance systems with active electrodes and small electrically shielded mobile EEG amplifiers has spawned a large increase in real-world EEG applications. Many of these systems are capable of high density (<128 channel) recording, but it is critical for the researcher to decide whether and to what degree an increase in the number of channels may result in a decrease in the quality of the recorded EEG (Luck and Kappenman, 2012). Importantly, the well-understood limitations of the spatial resolution of EEG limit the utility of high-density recording in ecologically valid environments (e.g., where measurement of EEG sensors co-localized in 3D space on a single-subject basis may be unfeasible). Moreover, with increasing channel density comes increases in the likelihood for poorly recorded or poorly monitored channels during recording. As such, if source-localization of underlying EEG/ERP generators is not a primary aim of the methodology (and we expect, in most applied cases it would not be), researchers may wish to record from a smaller density (e.g., 32 channels or fewer), at the benefit of better monitoring of data quality throughout the experiment.

On the theoretical side—researchers in human factors automotive research should carefully consider the linking hypotheses between specific electrophysiological indicators (e.g., P3b ERP amplitude, alpha power increases) and their purported cognitive interpretations. The ERP literature has a massive basic literature in which specific components have been very well-characterized relative to their eliciting conditions and underlying cognitive interpretations (Luck and Kappenman, 2012). One such example was reviewed earlier on characterizing the P3b under different states of distraction during driving. Limited work (e.g., Strayer et al., 2015) has attempted to examine ERP components in naturalistic settings. In future work, inventive approaches can be validated to use task-related responses or behaviors (such as eye-blink potentials or frequent vs. infrequent vehicle cues) as discrete events that can be recorded to estimate ERP components in real-world settings. At the same time, such characterizations in the spectral domain are not as clearly developed to date. However, this is changing, as basic research in cognitive electrophysiology shifts toward a more complete understanding of oscillatory mechanisms underlying human perception and cognition (e.g., Kahana, 2006), involving development in standardized analysis methods (Cohen, 2011), careful experimental characterization of specific oscillatory markers (e.g., alpha phase and perception, Mathewson et al., 2009; midline frontal theta and conflict resolution; Cavanagh and Frank, 2014), and the development of neurophysiologically guided models (Jensen and Mazaheri, 2010; Voytek and Knight, 2015). We expect that such development of basic research findings in cognitive electrophysiology will be a great asset in future applied research in contexts such as driving.

### Optical Imaging for Cerebral Blood Flow

#### Optical Imaging Quantification

*Optical imaging* methods allow for the visualization of the interaction of photons with tissues (Villringer et al., 1993). In recent years, there has been a rapid advancement in the application of non-invasive optical imaging methods such as functional near infrared spectroscopy (fNIRS) to study human brain and cognitive functioning. fNIRS is a neuroimaging method based on the principles of near-infrared spectroscopy, which was originally developed in humans for investigating clinical features of brain functioning (e.g., cerebral oxygenation; Jobsis, 1977). These principles have been extended to measure local changes in cerebral hemodynamic activity that can be used to infer information on the underlying neural activity due to neurovascular coupling, following similar logic to the Blood Oxygen Level Dependent (BOLD) signal in functional magnetic resonance imaging. NIR (700–1,000 nm) light is able to penetrate several centimeters through the skull and into brain tissue, allowing for non-invasive measurement of certain optical properties of cortical tissue. For example, changes in the concentration of oxy- and deoxy-hemoglobin can be measured via NIRS because oxy- and deoxy-hemoglobin have distinct absorption spectra that correspond to the different coloration of arterial and venous blood (Grinvald et al., 1986). These absorption characteristics make it possible to use a spectroscopic approach to measure changes in the concentration of oxy- and deoxy- hemoglobin as a function of neural activity, for example during cognitive task performance. In typical optical imaging systems, optical fibers, called optodes or sources, carry NIR light to the scalp while other optical fibers, called detectors, collect the photons as they emerge from the scalp. Each source–detector pair is a single channel. Multi-channel and wearable fNIRS systems have become commercially available with diverse montages capable of measuring brain activity across the entire scalp.

#### Optical Imaging for Cerebral Blood Flow in Driving Context

The application of fNIRS in driving research is in its infancy. Nevertheless, a number of interesting demonstrations of the utility of fNIRS for studying over-arousal states such as driver workload have emerged (e.g., Tsunashima and Yanagisawa, 2009; Liu et al., 2012, 2016; Sibi et al., 2016). For example, increases in oxygenated hemoglobin have been reported during simulated driving tasks under cognitive load compared to control conditions (Liu et al., 2012). A recent study (Unni et al., 2017) utilized fNIRS in a naturalistic driving simulator while doing a secondary task (modified version of 0–4 back). They found systematic increases in bilateral inferior frontal and temporo-occipital brain regions with increments in workload. Another study reported that fNIRS could be used to differentiate between low vs. high workload (n-back task) related hemodynamic activity in the prefrontal cortex while motorists drove in a realistic driving simulator (Herff et al., 2017). Furthermore, fNIRS have been used to monitor pilot's task engagement and working memory load in real-time (Gateau et al., 2015). On a related note, fNIRS have been found sensitive to increase in task difficulty in flight simulators (Causse et al., 2017) as indicated by an increased concentration of oxygenated hemoglobin and a decreased deoxygenated hemoglobin.

Other work has investigated effects of under-arousal related states with fNIRS. Research has related decreases in hemodynamic measures of cerebral oxygenation with fatigue in simulated driving (Li et al., 2009), and findings have been extended into actual highway driving (Yoshino et al., 2013). An increase in fatigue can be indexed by a decrease in cerebral oxygenation and mental stress can be indexed by an increase in cerebral oxygenation. Tsunashima and Yanagisawa (2009) examined changes in prefrontal activity via multi-channel frontal fNIRS systems in driving with and without adaptive cruise control. Their findings revealed substantial decreases in prefrontal activity when participants drove with adaptive cruise control relative to without, which was correlated with perceived workload (via the NASA-TLX). Similar decreases in activation of prefrontal cortex (lower cognitive load associated with drowsiness) were reported while participants monitored a simulated autonomous car driving task relative to higher prefrontal cortex activation during manual driving task (Sibi et al., 2016). Such findings indicate that optical imaging for cerebral blood flow is a valuable tool for assessing performance and neural efficiency in well-controlled realistic driving contexts.

#### Practical Considerations

One important limitation of fNIRS is that, because it relies on the measurement of absorption properties of light as a function of vascular changes in the brain, its temporal resolution is limited by the time-course of hemodynamic activity (on the order of seconds). In contrast, the development of recent ‘fast’ optical imaging methods, such as the event-related optical signal (EROS; Gratton and Fabiani, 2001, 2003), which measures scattering properties of light as a function of changes in neural activity, have a much higher temporal resolution (on the order of milliseconds). Although applications of this method in human factors research is sparse, fast optical imaging methods have growing promise. While the spatial resolution of optical imaging methods is higher than EEG, such spatial inference is constrained by the penetration depth of NIR light, which reaches only a few cm from the scalp surface. Therefore, imaging of activity from deep cortical and subcortical sources (beyond the outer cortical mantle) is limited. Recent work has also employed wearable fNIRS systems (Piper et al., 2014; McKendrick et al., 2016; Le et al., 2018) and simultaneous collection of fNIRS and EEG (Kassab et al., 2018), which can enable real-world monitoring in ecologically valid settings.

### Heart Rate (HR) and Heart Rate Variability (HRV)

#### Heart Activity Quantification

*Heart rate* (in beats per minute or bpm) is the number of heartbeats in 1 min (Jennings et al., 1981). Electrocardiography (ECG) is a well-established method to record the electrical activity of the heart. In psychophysiology, a lead II configuration (i.e., placing the negative electrode in the region of right collar bone, the ground near the left collar bone, and the positive lead over the lower left ribcage, or functionally similar variant) is commonly used to be able to record electrical activity of the heart via research grade equipment. A single heart beat wave in an ECG signal shows changes in electrical potentials (referred to as the P, Q, R, S, & T components and together they are referred to as the *QRS complex*, for review please see Berntson et al., 2007). The R component (one for each heart beat) is due to ventricular depolarization and for a lead II configuration, it has a larger magnitude and a sharper inflection than the rest of the components making it easily detectable. While heart rate is a count of beat per minute, *heart period* (also called inter-beat-interval) is the time in milliseconds between successive R spikes (Berntson et al., 2007). Heart rate is generally derived by converting mean heart period (in milliseconds) to heart rate (in beats per minute), see Berntson et al. (2007).

Heart data can also be collected via other technique including photoelectric plethysmography (PPG) and photoplethysmography imaging (PPGI). PPG technique includes use of a photocell (such as an infrared light-emitting diode) placed over an area of tissue with blood capillaries that is easily accessible (e.g., finger or ear lobe). Energy emitted from an infrared source passes through the tissue and reflects off the tissue. Changes in blood volume (due to heart beats) in an area can thus be assessed by the amount of light that was reflected back to the photodetector, and thus forms the basis of estimating heart beats (Berntson et al., 2007; Laborde et al., 2017). A similar concept is used in “wearables” which have photo-emitters and detectors placed on a convenient location (e.g., wrists and earlobes) making them easy to wear and collect data from them (Byrom et al., 2018; van Gent et al., 2018). This idea is used in vehicles with photo-emitters and detectors placed on the steering wheels, which allow collecting heart data (heart rate, HRV, and blood volume pulse) while driving. Another advancement in PPG is a contactless measurement technique called PPGI that detects color changes (e.g., the forehead area) in a video due to blood perfusions (Blöcher et al., 2017). Instead of photodiodes used in PPG, PPGI uses detector arrays in cameras to collect image sequences that contain information about bio-signals (e.g., blood volume pulse and respiration). Image and signal processing methods are utilized for beat-to-beat heart rate estimation (Blöcher et al., 2017; Madan et al., 2018).

On a related note, established guidelines for heart beat detection processing, with recommended parameters to derive heart rate and heart rate variability are provided in Jennings et al. (1981), Berntson et al. (2007), and Shaffer and Ginsberg (2017). Custom and open-source software has also been developed to automatically detect R peaks to calculate heart beats. As is true for most physiological measures, data should be visually checked to inspect the ECG data for artifacts and irregularities. Artifacts can be introduced in these data due to numerous reasons (such as motorists' excessive motion, sneezing and coughing, and irregular heartbeats) any of which can disrupt the ECG measurement or directly impact normal heart-beat patterns. Visual inspection helps insure that the heart beats are correctly marked by the detection software and physiologically improbable values are detected and then corrected.

*HRV* is variability in the time intervals of adjacent heartbeats (Berntson et al., 2007; Shaffer and Ginsberg, 2017). HRV can be derived from ECG data over a period of time ranging from short intervals (~1–5 min) up to longer intervals (~24 h). HRV metrics can be roughly categorized as falling under time-domain, frequency-domain, or non-linear measures of HRV (for a review see Shaffer and Ginsberg, 2017). Time domain-based parameters calculate the variations in heart beat intervals, such as standard deviation of R-R intervals (SDRR), percentage of successive R-R intervals that differ by more than 50 ms (pNN50), and root mean square of successive R-R intervals (RMSSD). A few time-domain parameters also represent geometric shape of R-R interval distributions, such as the HRV triangular index (i.e., plotting the integral of the ratio of RR interval density histogram by its height) and the baseline width of the RR intervals histogram (TINN), for details see Shaffer and Ginsberg (2017). Frequency-domain based measures transform the beat-to-beat variations in heart beat (R-R intervals) into frequency power bands via Fourier analysis (Task Force of the European Society of Cardiology, 1996). The most commonly used frequency-domain methods are low- and high-frequency power. A low-frequency (LF) power is the energy of heart rate oscillations in a lower-frequency (0.04–0.15 Hz) band. Similarly, high-frequency (HF) power is the energy of heart rate oscillations in a higher-frequency (0.15–0.4 Hz) band (Task Force of the European Society of Cardiology, 1996; Shaffer and Ginsberg, 2017). A peak in these frequency bands can also be calculated, which is an estimate of the peak frequency in the specific frequency band. Non-linear measures of HRV are useful in capturing the unpredictability and dynamic nature of heart rate time-series data (Shaffer and Ginsberg, 2017). Common measures include fitting an elliptical-shape to represent non-linear HRV and calculating approximate entropy (ApEn) and sample entropy (SmpEn), which characterize the complex pattern of time-series heart data (Shaffer and Ginsberg, 2017). Detailed discussions can be found elsewhere (Task Force of the European Society of Cardiology, 1996; Berntson et al., 2007; Laborde et al., 2017; Shaffer and Ginsberg, 2017).

#### HR/HRV in Driving Context

##### Over-arousal in driving context

Heart rate is a commonly measured index of physiological arousal in response to changes in driving demands. One of the most studied over-aroused cognitive states is workload. Numerous studies have examined changes in heart rate as a function of workload (Lenneman and Backs, 2009, 2010; Mehler et al., 2012; Heine et al., 2017). Heart rate was also found to increase while performing visual and auditory dual-tasks relative to single-task of driving in a simulator (Lenneman and Backs, 2009). Similarly, heart rate has been shown to be incrementally higher for systematically more difficult auditory dual-tasks while driving in a simulator (Mehler et al., 2009) as well as while driving on-road (Reimer et al., 2009). These findings of an incremental change in heart have been replicated in younger-aged (20–29 years old), middle-aged (40–49 years old), and older-aged (60–69 years old) adults (Mehler et al., 2012). Thus, heart rate increases with workload due to cognitive demand (Lenneman and Backs, 2009; Mehler et al., 2012; Ruscio et al., 2017; Hidalgo-Muñoz et al., 2018; c.f., Engström et al., 2005). Other efforts have also been made to utilize rhythmic and morphological parameters of a heart activity to explore mental workload. A recent study examined the influence of mental workload (due to a secondary task) on morphological parameters from ECG while completing a lane change task (Heine et al., 2017). They found that a combination of derived HR and HRV features (such as mean HR, RMSSD, pNN50, etc.) could be extracted from ECG data that could distinguish between workload levels and suggest that a combination of ECG features can be used to detect mental workload (for details see Heine et al., 2017).

Relative to HR, a fewer number of studies have examined HRV, especially in a systematic manner. HRV decreases with increasing task demands (Luque-Casado et al., 2016). HRV has been found to be sensitive to variations in attention levels while driving that may not be necessarily evident in driving performance (Lenneman and Backs, 2009) and thus HRV can have more sensitivity than behavioral measures. LF- and HF-HRV power bands are influenced by driving task (Zhao et al., 2012; Tozman et al., 2015; Wang et al., 2018). A study (Tozman et al., 2015) compared effect of demand levels (boredom, average demand, and high demand) on HRV in a driving simulator. Both LF- and HF-HRV varied for all the three conditions. High task demands reduced both LF-HRV and HF-HRV (Tozman et al., 2015). Some work has indicated that stress-inducing real-world driving tasks lead to increased heart rate and decreased SDNN, RMSSD, pNN50 (Lee et al., 2007). HRV also varies with workload experienced by drivers during simulated driving (Zhao et al., 2012; Heine et al., 2017; Hidalgo-Muñoz et al., 2018) and on-road driving (Lee et al., 2007). In addition, HRV variations due to cognitive workload have also been found in city traffic operators (Fallahi et al., 2016) and unmanned aerial vehicles operators (Jasper et al., 2016). HRV is sensitive to workload increases due to vigilance and situational awareness demands of the task (Saus et al., 2001; Stuiver et al., 2014; Jasper et al., 2016). However, at least one study (Shakouri et al., 2018) found no variation in heart rate variability metrics (RMSSD, LF, HF, and LF/HF ratio) as a function of higher traffic density while driving in a simulator, even though variations in subjective workload were found.

##### Under-arousal in driving context

HR and HRV are also sensitive to low-arousal states, such as vigilance and drowsiness. Decreases in vigilance over the course of a 3-h continuous driving task were indexed by a significant drop in heart rate over time (Schmidt et al., 2009). Drowsiness experienced in car drivers and aircraft pilots can also be associated with decreases in HR (Borghini et al., 2014). A recent on-road study (Biondi et al., 2018) found that driving a Tesla in semi-automated mode (e.g., autopilot) led to a lower heart rate relative to manual driving on a freeway. Another study found heart rate was sensitive to activity of the Adaptive Cruise Control (ACC) technology (Brouwer et al., 2017). Heart rate increased when ACC decelerated more suddenly compared to instances when the car decelerated more gradually (Brouwer et al., 2017). These findings suggest that heart rate is a sensitive measure that can assess cognitive processing pertaining to advanced technology in semi-autonomous vehicles.

Other studies have found that LF-HRV and HF-HRV vary with fatigue (Liang et al., 2009; Sugie et al., 2016). A recent study (Wang et al., 2018) found that changes in fatigue levels while driving can be represented by non-linear measures of HRV (e.g., sample entropy). Variations in drowsiness levels can also impact HRV (Noda et al., 2015; Piotrowski and Szypulska, 2017). Another recent study found that variations in HRV (TINN and RMSSD) was higher when participants drove a vehicle in automated mode relative to the manual mode (Biondi et al., 2018). Perhaps, drowsiness and a lack of engagement in the driving task during automated mode may have led to a higher HRV. HRV and blink rates have also been shown to assess sleep onset (Noda et al., 2015). HRV-based assessment algorithms can be used for early detection of fatigue and drowsiness to augment attention and performance (Patel et al., 2011; Zhao et al., 2012; Abe et al., 2016; Vicente et al., 2016).

#### Practical Considerations

Heart rate and its variability are inexpensive and reliable measures that are relatively easy to record with research-quality equipment that meets recommended guidelines (Task Force of the European Society of Cardiology, 1996). It has good signal to noise ratio as well (R-R peaks can be detected even in very noisy environments). Consequently, it is also not difficult to collect in lab as well as in unpredictable field studies, especially with the availability of mobile data recording systems. However, these advantages can also lead to misuse of this methodology. Great attention to the data collection and processing are required to have meaningful data. Skin preparation (e.g., cleaning with alcohol wipes) before electrode placement and signal monitoring to collect good quality data can drastically reduce post-processing (e.g., Berntson et al., 2007). Participants should be comfortably positioned to avoid physiologically induced changes in heart rate such as altered breathing rate due to postural adjustments. Body movements should be minimized and accounted for as such movements can add noise and also add movement-related heart rate changes. Effective data cleaning to remove artifacts and noise are a must, otherwise heart data will be uninterpretable.

Some recording devices do not utilize the traditional QRS complex from an ECG to calculate HR and HRV. For example, PPG uses a photoelectric sensor that estimates changes in blood volume to calculate HR. There are a few methodological challenges that should be considered before adopting such PPG-based systems. PPG records a lagged cardiac response further away from the heart (e.g., from fingers and earlobes). Unlike ECG based estimates that have a sharp spike for the R component, PPG-based methods instead show a less pronounced curved peak of the blood volume pulse signal, which makes accurate and automatic detection of heart period relatively more difficult (Laborde et al., 2017). Moreover, ECG-based estimates of HR and HRV are recommended for more reliable results because it allows visual inspection and artifact correction of heart data. Such methodological differences between PPG and ECG can explain why PPG and ECG findings are comparable during rest, but are not comparable during stress, for example (Schäfer and Vagedes, 2013).

On a related note, commercialized equipment meant for exercise and fitness tracking fail to meet established guidelines for heart data collection and processing (e.g., minimum sampling rate and access to raw data for necessary artifact correction methods), which are necessary to make meaningful interpretations (see Berntson et al., 2007; Quintana et al., 2016; Shaffer and Ginsberg, 2017). Similarly, smartphone camera-based assessments have methodological challenges, including very poor sampling rate, illumination variation (due to confounds like weather and time of day), poor signal-to-noise ratio, and motion-related artifacts that can lead to inaccurate interpretations (Laborde et al., 2017; cf., Nowara et al., 2018; van Gent et al., 2018). Ensuring the validity and inter-device variability of wearables (which utilize a PPG-based or camera-based HR system) with an established ECG-based equipment is a necessary step to be able to validate data collected from wearables. However, most commercialized equipment has not been validated in such a manner (Quintana et al., 2016). Without this critical validation step, data collected from commercialized non-research grade equipment does not have convergent validity and should be discouraged by the scientific community until such standards are met. While innovation is critical to be able to collect psychophysiological data in real-world settings, careful adoption and cross-checks with existing gold standards are necessary to make meaningful progress in the adoption of these technologies in real-world driving research.

Moreover, HF-HRV has been found to be impacted by parasympathetic nervous system, however, LF-HRV is influenced by both sympathetic and parasympathetic nervous systems (Berntson et al., 2007; Laborde et al., 2017). Thus, LF-HRV should not be described as a metric of sympathetic activity, but instead be interpreted as a mixture of sympathetic and parasympathetic influences. On a related note, the LF/HF ratio has been a controversial metric as it assumes that LF is due to sympathetic activity while HF is due to parasympathetic (Billman, 2013). The LF/HF ratio was originally based on 24 h recordings, while shorter duration recordings (even 5 min long) have also been calculated. The duration of recording (e.g., 5 min vs. 24 h) can also lead to uncorrelated findings and some metrics are better for short term recordings than others (Shaffer and Ginsberg, 2017).

Another metric we would like to highlight is heart period. Heart rate and heart period have been used interchangeably, however in some instances heart period may be a better choice. Even though, heart rate is more commonly used metric, use of heart period instead of heart rate is recommended measure of autonomic activity because heart period changes more linearly over time (Quigley and Berntson, 1996; Berntson et al., 2007). Heart period should specially be used when comparing changes in heart activity due to experimental manipulation or due to between group differences for short time periods. Further information on heart activity related metrics can be found in detailed reviews (Jennings et al., 1981; Task Force of the European Society of Cardiology, 1996; Berntson et al., 2007; Laborde et al., 2017; Shaffer and Ginsberg, 2017).

Not all heart-based metrices may be sensitive to the variations in cognitive state during driving task. For instance, a study compared several commonly used metrices for HR and HRV cognitive workload during highway driving (Mehler et al., 2011). While HR was robust in differentiating between cognitive workload in single vs. dual tasks, HRV indices were less robust (e.g., smaller effect sizes). A few HRV indices varied with workload (RMSSD, SDSD, and LF power), however others (SDNN, NN50, pNN50, HF power, and LF/HF) did not significantly differ with workload (Mehler et al., 2011). These findings suggest that depending upon the task, certain indices may be more sensitive to variation in cognitive state than other indices that may be less robust.

In addition, researchers should consider other contextual factors that may vary across participants and may confound study interpretations. A confounding factor that can potentially bias HF-HRV comparisons between conditions of interest is differences in respiration (Grossman, 1992; Berntson et al., 2007; Laborde et al., 2017). Respiration related-parameters should be accounted for by using them as covariates with such HRV indices (for a detailed discussion, see Berntson et al., 2007; Laborde et al., 2017). Similarly, other factors may impact HR/HRV, including task characteristics and motorists' state (relaxation, engagement, and motivation) and activities (smoking and posture). For instance, HRV may increase over time if the task becomes less difficult over time, which may put motorists in a more relaxed state (Jasper et al., 2016). Similarly, HRV may also increase over time with disengagement or demotivation to perform a difficult task (Jasper et al., 2016). Careful consideration of contextual factors will afford accurate and reliable measurement of HR/HRV indices in applied driving settings.

### Blood Pressure (BP)

#### BP Quantification

*BP* (in millimeters of mercury, also written as mmHg) is the force exerted against the walls of the blood vessels (Shapiro et al., 1996; Berntson et al., 2007). Depending upon the stage of the dynamic cardiac cycle, BP differs from lowest to highest levels. During a single cardiac cycle, *diastolic BP* is the lowest level of arterial pressure when the heart is filled with blood and *systolic BP* is relatively the highest level of arterial pressure (Shapiro et al., 1996; Berntson et al., 2007). As invasive methods to record BP require additional safeguards and equipment, most psychophysiology research studies focus on non-invasive approaches to record blood pressure. Three relatively non-invasive methods are auscultatory or oscillometric methods, arterial tonometry, or the volume-clamp methods (see for details, Berntson et al., 2007). The most common method is auscultatory measurement, which records the sounds of blood flow by placing a cuff on the upper arm and a stethoscope placed over the brachial artery to identify the systolic and diastolic blood pressure (Shapiro et al., 1996; Berntson et al., 2007). Physiological arousal during mentally effortful situations leads to greater vasoconstriction and cardiovascular reactivity evidenced by increased heart rate and blood pressure and decreased heart rate variability (Lundberg et al., 1994; Ottaviani et al., 2016). BP increases with psychological stress (Ottaviani et al., 2016) and is correlated with self-reported stress (Lundberg et al., 1994). However, cognitive workload may not reliably influence BP (ElKomy et al., 2017).

#### BP in Driving Context

Limited research has examined over- and under-arousal via BP in driving contexts. Systolic BP and BP variability have been found to increase while driving in simulated high traffic conditions that had high workload demands (Stuiver et al., 2014). Fatigue was also associated with a decrease in systolic BP and HR (Liang et al., 2009). However, other studies have not found a reliable effect of stress on BP (Simonson et al., 1968; Littler et al., 1973; Lee et al., 2007). One study found no significant change in BP from beginning to end of the drive with a short period of arterial pressure changes during events such as overtaking that returned to baseline (Littler et al., 1973). BP was also not found to vary in an on-road stressful driving task speed in a simulator even though HRV parameters were significantly impacted (Lee et al., 2007).

Nevertheless, BP is a very useful measure to understand the factors that impact driving performance. One clear example of this comes from a simulator-based study investigating aggressive driving behavior in irregular traffic flow and under time pressure (Drews et al., 2012). Irregular traffic patterns were not found to impact BP. However, male drivers who were under time pressure to drive faster in order to receive a monetary incentive, had elevated systolic BP compared to females under time pressure or compared to male drivers who were not under time pressure. In fact, females did not show any elevated blood pressure under time pressure (Drews et al., 2012). These findings suggest that individual difference factors such as sex differences and motivation to drive aggressively may impact driving behavior and associated physiological signals. Other studies have shown that trait-level variation in BP (such as a history of high BP i.e., hypertension) is an important measure to capture health and age-related impact on driving performance in vulnerable older populations (Lyman et al., 2001; Siren et al., 2004). A 5-year longitudinal study that examined the effect of urban bus driving on BP found that the number of hours driven per week predicted higher diastolic BP (Johansson et al., 2012), suggesting that there are cumulative effects of cognitive demands and stress of continuous driving.

#### Practical Considerations

While heart-rate was reported to rapidly change in response to car racing, BP was “less responsive” (Simonson et al., 1968). Other studies have found that BP does not change significantly during on-road driving (Littler et al., 1973; Lee et al., 2007). A few BP recording-related reasons could play a role. BP can rapidly change over time so multiple readings are recommended for a more accurate estimate. However, a limiting factor is the BP equipment. The pressure from a cuff worn by the responder can become uncomfortable and disruptive within a few minutes. Continuous reliable BP measurement (especially via volume-clamp) is uncomfortable, distracting, and potentially disruptive to driving. This limits the frequency of samples that could be collected, which are about 1 reading per minute. Also, the BP recordings are sensitive to movement so in an on-road study, it is less feasible to accurately record multiple BP reading from participants while drivers are actively involved in the driving process. While some alternative methods to record blood pressure (e.g., plethysmography) may be available, methodological issues similar to those discussed in recording heart activity apply to BP as well and it is crucial to evade poor quality unreliable equipment. In sum, BP provides valuable insights about vulnerable states of the drivers, however, in a real-world driving context, methodological concerns can limit reliable data collection. Much future work is required to be able to measure reliable and non-invasive BP activity.

### Electrodermal Activity (EDA)

#### EDA Quantification

*EDA*, previously known as galvanic skin response, is a change in electrical potentials of the skin that can be used to make interpretations about the psychological phenomena of the responder (Boucsein et al., 2012). EDA can be measured via exosomatic or endosomatic techniques. Exosomatic techniques—a more commonly used method used in applied research—apply a small current through a pair of electrodes and then measure electrical resistance (or its reciprocal, i.e., conductance) from the skin. Because the current is kept constant, it is possible to measure changes in the voltage between the electrodes that will vary directly with changes in skin resistance, following Ohm's lab (see Dawson et al., 2007 for a technical review). Endosomatic techniques measure passive changes in intrinsic electrical activity without application of an external current. For details on EDA recording techniques, see Fowles (1986), Dawson et al. (2007), and Boucsein et al. (2012). Higher EDA is indicative of physiological arousal due to increased sympathetic autonomic nervous activity (Dawson et al., 2007; Lohani and Isaacowitz, 2014). EDA is sensitive to physiological reactivity and many other factors, such as respiration and mental effort (Dawson et al., 2007). Commonly derived EDA metrics (Dawson et al., 2007; Boucsein et al., 2012) include slowly varying tonic level of electrical conductivity (skin conductance level; SCL) and phasic increase in magnitude electrical conductance in response to an unexpected or relevant event (skin conductance response; SCR). Non-linear EDA metrics that can differentiate between increased cognitive load vs. recovery phases of stressors have been identified as well (Visnovcova et al., 2016).

#### EDA in Driving Context

In driving research, systematic variation in several arousal-related constructs can impact EDA. Most commonly investigated is cognitive workload. SCL is higher during increased workload in dual-task relative to single-task driving (Mehler et al., 2012). A systematic investigation of workload increments in one on-road driving study (Mehler et al., 2012) found a systematic increase in SCL as a function of three levels of auditory workload secondary tasks relative to single driving task for young, middle, and older age groups. These findings suggest that SCL can be used to index workload levels in driving context. High SCR has also been found to increase with workload experienced by motorists while driving on difficult road types that required avoiding more traffic and making more decisions (Schneegass et al., 2013). A recent study reported SCR amplitude increased with cognitive load due to dual-task driving (Ruscio et al., 2017). Additional workload experienced due to texting and navigation (Seo et al., 2017) and speeding (Kajiwara, 2014) while simulated driving was also found to increase EDA.

EDA also varies with other physiological arousal-related constructs. EDA based indices can be used to detect stressful events during driving (Affanni et al., 2018). A recent study utilized feature extraction and discrimination processing techniques to classify EDA data into low, medium, vs. high stress levels with about 82% recognition rate (Liu and Du, 2018). Another recent study found higher SCLs when participants drove a simulated vehicle in autonomous mode compared to manual mode (Morris et al., 2017). Higher skin conductance levels could be indicative of lower levels of trust in the autonomous mode than manual mode. State anxiety during simulated driving was also found to be associated with SCL (Barnard and Chapman, 2018). Another recent study found that relative to sleepiness, higher skin conductance levels are found during wakefulness, effects which are indicative of comparatively higher sympathetic activity (Schmidt et al., 2017).

#### Practical Considerations

In driving contexts, EDA is shown to vary due to many cognitive states, such as workload, stress, anxiety, sleepiness, all of which are influenced by sympathetic nervous system activity. This allows the use of EDA in assessment of various psychological phenomena (Dawson et al., 2007). Therefore, caution should be exercised while interpreting changes in EDA in an applied and less-controlled setting as it is sensitive to not one, but many psychological variables. In the driving context, careful choice of filters to remove artifacts (Affanni et al., 2018) and identification of cognition-related features (Chen et al., 2017; Liu and Du, 2018) that have been successfully implemented could be utilized to improve accuracy and detection. One disadvantage of EDA is that it has a slower response (lag of 1–3 s) after the stimulus has occurred (Dawson et al., 2007). In instances when near-real time physiological responses need to be detected, EDA may be relatively slower (than cardiovascular measures). Another point to consider is that, similar to other physiological measures, not all individuals have the expected skin conductance response (Dawson et al., 2007). This is another reason to avoid reliance on a single measure, but multiple channels, to capture the psychological phenomena of interest.

### Electromyography (EMG)

#### EMG Quantification

*EMG* is used to measure the electrical activity generated by muscle fibers (Fridlund and Cacioppo, 1986; van Boxtel, 2001). Surface EMG is captured by placing small surface electrodes on specific muscles of interest, which is then digitized and amplified to record muscle activity (Fridlund and Cacioppo, 1986). Numerous features can be extracted from the EMG signals. Root mean square of the signal (in microvolts) is a recommended and commonly reported EMG signal amplitude (Fridlund and Cacioppo, 1986). Other commonly assessed statistical features are peak spectral density, peak amplitude, and peak frequency. A specific muscle's activity can provide insights into the psychological processes underplay. For instance, the smile muscle (or zygomaticus major) and the frown muscle (or corrugator supercilii) have been used a lot in emotion research to identify positive and negative behavioral expressions. For example, more frown muscle activation can be an index of negative behavioral expressions (Lohani and Isaacowitz, 2014; Lohani et al., 2018). Psychological processes (e.g., stress) can lead to sympathetic nervous system activity (Lundberg et al., 1994), which can elicit muscular tension. Researchers have studied muscular activations under controlled conditions to index mental processes (Lundberg et al., 1994; Wijsman et al., 2013; Luijcks et al., 2014). Applied driving research has successfully assessed psychological processes by assessing EMG (Healey et al., 1999; Fu et al., 2016; cf., Morris et al., 2017; Ma et al., 2018).

#### EMG in Driving Context

In driving contexts, surface EMG has been utilized to study psychological and physiological stress (Jonsson and Jonsson, 1975; Wikström, 1993; Balasubramanian and Adalarasu, 2007; Ahlström et al., 2018). Stress and fatigue have been studied by recording electrical activity from relevant muscles. For instance, variations in the trapezius muscle (a major back muscle that extends from the neck to shoulder blades and lower spine) and deltoid (triangular muscle located on uppermost part of an arm and the top of shoulder) are influenced by mental stress (Wikström, 1993; Balasubramanian and Adalarasu, 2007; Hirao et al., 2007; Wijsman et al., 2013; Luijcks et al., 2014; cf., Morris et al., 2017). A recent study (Lee et al., 2017a) recorded trapezius muscle activity to detect stress in a driving simulator under relaxed and stressed conditions. A continuous increase over time in muscular tension was associated with greater stress experienced due to driving task (Lee et al., 2017a). Muscular tension can thus be a useful metric of stress level that can be utilized in driving research.

It is worth noting that muscular fatigue and discomfort are not isolated issues (Leinonen et al., 2005) and they cause psychological distress and disrupt cognitive performance while driving. Muscle fatigue while driving has been studied by examining changes in muscular tension in shoulder and neck muscles (Sheridan et al., 1991; Wikström, 1993; Balasubramanian and Adalarasu, 2007; Hirao et al., 2007). Compared to the beginning of the drive, continuous driving can lead to reduced back muscles (e.g., trapezius and deltoid) activity and fatigue. Muscular fatigue (measured by EMG of back muscles) is associated with decreases in power of EMG activity-related frequency band (Hostens and Ramon, 2005; Balasubramanian and Adalarasu, 2007; Hirao et al., 2007). Surface EMG is a helpful way of identifying discomfort in fatigued and weak muscles and targeting rehabilitation for skeletomuscular problems specially in professional or long-distance drivers (Balasubramanian and Adalarasu, 2007). A recent study (Artanto et al., 2017) has also used a low-cost EMG system to detect drowsiness. An EMG sensor attached to muscles around eyelid region captured the duration of eyelid closure as an indicator of drowsiness (Artanto et al., 2017). Another recent study has proposed a system that can detect real-time changes in EMG (Mazzetta et al., 2018). Further research is needed to validate EMG's applicability in real-world settings.

#### Practical Considerations

EMG measurement enable recording continuous data from the specific muscle of interest without obstructing the driving task. Such objective information can be helpful in learning about muscular activity (and relevant cognitive states) that may not be necessarily visible to the researchers or under the awareness of the responder. However, it is essential to pay attention to any outliers or irrelevant events that may add noise to the EMG signal and impact signal interpretation. Irrelevant events can include muscular activity due to driving-unrelated (e.g., continuous posture change, scratching skin, or touching the electrodes) and driving-related (e.g., functional steering activity) movement and yet unrelated to the cognitive state (e.g., mental workload) of the driver (Mehler et al., 2009). In real-world settings, it can be tedious to tease apart muscular activity due to other confounding reasons from activity relevant to changes in cognitive states. Furthermore, the task under investigation is also of importance. For instance, a study that compared muscular tension while driving car autonomously vs. manually found no differences in EMG signals, but significant differences were found for SCL (Morris et al., 2017). This suggests that for some tasks the muscular activity may not significantly differ, but may still be psychologically different in other modalities. This also highlights the importance of multiple measures.

### Thermal Imaging

#### Thermal Imaging Quantification

The measurement of changes in skin temperature is a useful technique to detect and track attributes of a responder, such as body posture and emotional expression (Gade and Moeslund, 2014; Rai et al., 2017). A special merit of this technology is that it enables sensing the real-time state of motorists non-invasively without disrupting driving related tasks. In addition, unlike RGB cameras, thermal cameras do not depend on an external illumination (Gade and Moeslund, 2014; Rai et al., 2017). Objects that emit radiations in the mid-to-long wavelength infrared spectrum (3–14 μm), such as the human body (but not inanimate objects) can be detected via thermal imaging (Gade and Moeslund, 2014; Rai et al., 2017). Changes in temperature distribution, as captured by the thermal cameras, are utilized to make meaningful interpretations. For instance, facial thermography can be used to capture the heat distribution in facial locations known to vary with sympathetic activity as a metric of the varying psychological phenomena. Most commonly investigated facial locations include the forehead and nasal temperature changes.

Sympathetic autonomous nervous system activation may lead to constrictions of blood vessels, thereby decreasing temperature in extremities, such as the nose (Or and Duffy, 2007; Gade and Moeslund, 2014). For example, mental workload changes lead to temperature variations in the forehead, nose, cheeks, and chin regions (Stemberger et al., 2010; Marinescu et al., 2018). A recent study examined the validity and sensitivity of thermal imaging in assessing variation in cognitive load (Abdelrahman et al., 2017). Increased cognitive task difficulty led to significant increases in the forehead temperature and decreases in nose temperature (Abdelrahman et al., 2017). The largest effect sizes were found when the difference in forehead and nose temperature was estimated. Higher task difficulty led to an increase in forehead and nose temperature differences (Abdelrahman et al., 2017). Additional work has also examined real-time sensitivity of thermal imaging and found that specialized thermal cameras can detect changes in cognitive load with a latency of 0.7 s post eliciting event (Abdelrahman et al., 2017). This finding suggests that this methodology has a high relevance for real-time assessments of cognitive load in applied settings like driving.

#### Thermography in Driving Context

In driving contexts, facial thermography was found to be useful in assessing over-arousal constructs such as mental workload (Or and Duffy, 2007; Murai et al., 2008). Performing a secondary workload task (mental arithmetic) while driving in a simulator as well as an on-road car led to a decrease in nasal temperature with stable forehead temperatures (Or and Duffy, 2007). Drop in nasal temperature also correlated with self-reported workload (Or and Duffy, 2007). Another study found increases in the difference between nose and forehead temperature increased with mental workload (Kajiwara, 2014). Participants' nasal temperature varied as a function of mental workload in simulated driving (Kajiwara, 2014). Workload variation indexed by changes in nasal temperature were also reported during ship navigation using a simulator (Murai et al., 2008), highlighting its utility in applied settings.

Furthermore, facial thermography can be useful to examine and infer heat distribution in faces during emotional states. This method could be promising and may provide a non-invasive approach to capture emotional states because current methods of emotion recognition using facial features detection software have limitations. One study used an infrared thermal camera to non-invasively detect face regions and recognize emotional states of motorists (Kolli et al., 2011). This study suggests that thermography can improve face detection algorithm for in-vehicle settings thereby facilitating ADAS.

In another line of work (Cheng et al., 2007), a combination of thermal infrared and color cameras have shown to be effective in sensing body movements in real-time on-road driving. Similarly, infrared streaming has been used to develop posture and occupancy sensory systems (Kato et al., 2004; Trivedi et al., 2004). Another recent study reported successful use of near-infrared light and thermal camera sensors to identify aggressive driving behavior (Lee et al., 2018) and were able to categorize aggressive driving from relaxed driving. The above studies suggest that thermography has the potential to be a useful non-invasive technique that can be validated to capture cognition-relevant states and improve traffic safety.

#### Practical Considerations

Thermal cameras are used in numerous industrial, agricultural, and military settings (Gade and Moeslund, 2014). They can be extremely useful in vehicular technology because they are non-contact sensors and can work regardless of external illumination. Nevertheless, further testing is needed to better understand how this technology would improve our understanding of cognitive states in traffic safety. Further systematic investigation and replication of thermography as a function of cognitive workload, stress, and drowsiness after controlling for confounding factors, such as environmental factors (e.g., weather conditions and air conditioning), are needed to be able to make confident assessments of cognitive states. The results so far look promising.

### Pupillometry

#### Pupil Quantification

Pupillometry is the measurement of pupil size and reactivity. Modern pupillometry is measured via optical eye-trackers that use some combination of monitoring infrared light reflections from the cornea, the back of the lens, and the pupil, as well as absorption of light by the pupil (e.g., dark-pupil tracking). Most modern eye-tracking devices can monitor pupil location (and eye-fixation location) with very high resolution (>1,000 Hz) non-invasively and at a substantial distance from a participant. Thus, measurement can occur in highly ecologically valid environments, without participants having to make any overt responses. Since the 1960's it has been shown that pupil dilation changes as a result of mental activity—for example, increases in arousal and cognitive workload (e.g., Hess and Polt, 1964). In a classic study demonstrating the sensitivity of pupillometry to cognitive demands, Kahneman and Beatty (1966) showed that pupil dilation increases parametrically with an increasing number of words to recall in a simple word list memory task. Moreover, they showed that this increase in workload persists over a maintenance interval, and reduces parametrically as each word is retrieved (and released) from memory. These findings, along with a number of other demonstrations of pupillary sensitivity to cognitive workload, for example in math problem solving (Sirois and Brisson, 2014), working memory and individual differences in intelligence (Tsukahara et al., 2016), aging and verbal memory load (Piquado et al., 2010), has led to wide interest in this measure as a physiological marker of arousal and cognitive effort.

Janisse (1977) remarked that the eye is the only “visible part of the brain.” Indeed, detailed models of the neurophysiology of pupillomotor functioning are developed and growing, including an understanding of the innervation of the sphincter and dilator muscles by the autonomic nervous system (Miller et al., 2005), as well as the neuromodulatory relationship between pupil dilation, activity in the locus-coeruleus (LC; a neuromodulatory nucleus in the dorsal pons of the brainstem strongly linked to phasic and tonic arousal, cognitive control, and monitoring functions), and norepinephrine (Gilzenrat, 2006). For instance, a high correlation (0.6) between spike frequency and pupil diameter has been found, whereby large pupil diameter equates to high LC activity (Rajkowski et al., 1994). Demberg (2013) have also recently reported changes in pupillometry due to linguistically induced cognitive load (e.g., comprehending syntactically demanding sentences). Other recent work has also examined user state related changes in pupil diameter in lab-settings such as variations in valence and arousal (Kassem et al., 2017) and interest in real-time (Jacob et al., 2018).

#### Pupillometry in Driving Context

Eye-tracking has been used extensively in studying visual perception and attention in driving contexts, however the unique use of pupillometry as an index of real-time physiological indicator of cognitive workload is only lately growing in popularity (Schwalm et al., 2008). For example, Cegovnik et al. (2018) recently validated a low-cost eye-tracker and showed that pupil dilation increases with increments in cognitive load due to a secondary memory task (n-back) (see also Recarte and Nunes, 2000 for similar results). Pupillometry has also been adopted in driving research while motorists drove in a simulated driving context. Pupil diameter was found to reliably increase with increases in cognitive load (Palinko et al., 2010; Faure et al., 2016). Other work has use machine learning algorithms to detect cognitive load while driving from pupillometry data (Yoshida et al., 2014). A recent study found that during simulated driving, pupil dilation could detect increases in cognitive load imposed by a secondary task within a lag of 1 s (Prabhakar et al., 2018). This suggests that pupillometry could be used as a near-real time index of cognitive load.

Pupillometry has also been used to differentiate between alertness and drowsiness (Soares et al., 2013). Alertness is associated with increased mean pupil diameter and decreases in standard deviation (i.e., stable), whereas drowsiness is associated with decreases in diameter, but increases in standard deviation (i.e., fluctuations) in pupil diameter (Morad et al., 2000; Wilhelm et al., 2009). Fluctuations in pupil size have been proposed to be a reliable index of drowsiness-related impairment while driving (Maccora et al., 2018). Pupil dilation was also found sensitive to fatigue levels while driving with a decrease in fatigue being associated with an increase in pupil diameter (Schmidt et al., 2017). Although early, these findings, along with others (for a recent review see Marquart et al., 2015; Maccora et al., 2018) suggest that pupillometry is an efficient, ecologically valid, and low-cost physiological reporter variable for indexing cognitive states in driving in highly-controlled environments like realistic driving simulators.

#### Practical Considerations

In lab settings, pupil diameter was found to be a reliable, non-invasive, and real-time measure of workload (Marinescu et al., 2018). However, in on-road settings, it is quite challenging to capture interpretable pupil information due to large variations in luminance that are hard to control across conditions and participants. Indeed, photopupillary reflex is massive in magnitude relative to changes in pupil size related to cognitive and attentional factors. As such, if there are considerable changes in lighting conditions (e.g., sunny vs. cloudy days), this can create considerable noise in the pupillary signal. Moreover, if specific conditions of interest are confounded with respect to overall luminance (e.g., driving during the day vs. driving at night), this overall pupillary light reflex-related shift should be taken into consideration. Furthermore, if investigating event-related pupillary responses in driving, one should be careful to determine that differences in pupil dilation are not only due to differences in visual stimulation (e.g., presenting a luminant STOP sign). Modeling techniques have also developed methods to infer cognitive workload after accounting for some variations in lighting conditions (Pfleging et al., 2016; Reilly et al., 2018).

Marshall (2002) have developed a signal processing method for extracting high-frequency changes in pupil dilation that they argue is uniquely related to cognitive components (*Index of Cognitive Activity* or ICA). However, this method is a commercially available “black box” system, and should be interpreted with caution given that the exact algorithm used to calculate ICA from raw pupillometry is not open source. Other work has estimated an *Index of Pupillary Activity* (IPA) inspired by ICA, that uses wavelet-based algorithms to decompose pupil data (Duchowski et al., 2018). IPA was found to differentiate between low vs. high mental workload (Duchowski et al., 2018). Another important feature to consider is that measurement of pupil dilation is affected by eye-movements and relative gaze position (e.g., Gagl et al., 2011). When gaze position changes from central to peripheral locations, the recorded pupil shifts from a circular to an elliptical shape from the point of view of fixed camera location. This change in the recorded geometry of the pupil is accompanied by changes in overall pupil size, irrespective of actual changes in dilation or constriction. Gagl et al. (2011) have developed methods for the measurement and removal of such systematic influences. Nevertheless, researchers should be careful to measure gaze position and to design studies such that likely visual target locations are not confounded across conditions of interest.

## Challenges and Recommendations

Psychophysiological research has made tremendous progress in developing methods to quantify cognitive processes. Most of this research has been conducted in carefully controlled environments to be able to interpret with certainty what changes in a physiological signal may imply about the psychological phenomena under investigation. Physiological signals are valuable to understand how people interact in real-world contexts. Driving research is an excellent application of psychophysiological methods to understand and interpret how people interact with automation in natural settings, which in turn can inform intelligent systems to improve driving performance and safety. As evidenced by much of the growing research base discussed above, psychophysiological measures can be successfully adopted to meet these goals. At the same time, lack of adherence to research protocols and guidelines can seriously jeopardize meaningful use of these methodologies. Here we highlight a few general challenges and recommendations that cut across all psychophysiological measures in driving research when collecting data from real-world driving settings—which are less predictable than lab settings— to improve data-quality and aid in effective interpretation.

### Valid and Reliable Quantification of Construct

Depending upon the task and setting (lab-based simulator or field study), some physiological measures will be more suitable and feasible than others. For example, in a simulator with very controlled body movement, continuous blood pressure using the volume clamp method can be collected. However, while on-road, this equipment may compromise drivers' safety and thus is not feasible. Other measures like ECG and thermal cameras are highly mobile and feasible. Careful observations can allow interpretation of cognitive processes while driving. One important concern is the possibility of misinterpreting the relationship between physiological signals and cognitive processes (Cacioppo and Tassinary, 1990; Cacioppo et al., 2007). Often, physiological measures (such as HR, EDA, EMG) are impacted by multiple processes, such as drowsiness, stress, and workload, which can lead to interpretive caveats. Systematic variations in different experimental conditions can help tease apart the underlying mechanism causing autonomic activations to be able to draw clear inferences. However, in an applied setting like driving a car in unpredictable traffic, control over the experimental task is largely out of the control of the researcher. Confirmatory independent measures are important to validate the construct of interest in the study. Similarly, it is helpful to ensure that the construct of interest reliably varies across conditions and that the experimental manipulation was effective.

### Individual Differences

A combination of factors may influence physiological signals, including trait-level variables such as demographic factors (age, gender), task experience (professional, experienced, inexperienced), anxiety, and certain health conditions and medications (e.g., cardiovascular health). State-level variations such as stress-levels unrelated to task, caffeine intake (which may change autonomic activity), and engagement/motivation and frustration during the task can also interact with individual differences in ways that may not be readily apparent. Combining data from participants after considering such trait- and state-level variables can help in proper interpretation of study findings.

On a related note, a critical challenge in multi-modal recordings is that individuals may be highly reactive as assessed by one measure but not necessarily, according to another. There is considerable variability across individuals in how closely physiological, behavioral, and subjective measures covary over time with one another (Lohani et al., 2018). Furthermore, it is possible that only some individuals may be sensitive to the experimental manipulation (Drews et al., 2012). Such individual differences may lead to variations in psychophysiological assessments and may also explain to some extent lack of significant differences across experimental conditions. Many, if not all, of these measures are currently utilized within paradigms where we are studying relative changes in the outcome across conditions (e.g., P3b amplitude is a difference wave, HRV% change, %signal change in BOLD response, etc.), for which these measures do not have currently well-understood absolute thresholds for making strong absolute judgements. While there isn't a fixed threshold for physiological measures that can be used across individuals to define high and low arousal levels, relative changes from baseline can be a useful way of assessing variations in arousal levels from optimal levels for the individual. If the system can be calibrated on what is a “normal” range for an individual, then significant variations from this calibrated range can be a way to detect sub-optimal arousal levels.

### Baseline Assessments

Baseline assessments provide insights about the physiological state of the responder when the experimental condition was absent. It also allows to control for physiological activity due to any prior conditions, so that the change in the experimental condition of interest is interpreted relative to the state right before the condition started. A single baseline is generally not enough, especially when there are multiple conditions. It is a good practice to capture as many baseline assessments and as close to the experimental condition as possible. Another alternate design to consider (for measures with high temporal resolution) is an event-related design, where activity is time-locked to specific events of interest. In this design, pre-event activity in the measure is subtracted from the overall physiological time series, resulting in a strong baseline control for each trial (e.g., ERPs).

### Sampling Rate, Filtering, and Signal Quality

Nearly all physiological signals discussed above are analog signals, which have to be digitized for further processing. Choice of optimal sampling rate and filtering helps avoid signal distortions (Jennings and Allen, 2016), and as such, knowledge of signal processing characteristics of the target physiological measures is necessary for researchers to effectively use these tools. Optimal sampling rate differs by the physiological signal's frequency characteristics, and poor sampling rate can distort waveform characteristics, and induce artificial oscillatory characteristics that are not part of the true analog signal (i.e., aliasing). For example, for HRV analysis, the recommended sampling rate is at least 250 Hz (Task Force of the European Society of Cardiology, 1996). Some commercial wearables (e.g., fitness-related wrist watch sensors) have sampling rate as low as 60 Hz, which will lead to signal aliasing (Jennings and Allen, 2016) and inaccurate and uninterpretable HRV values. The sampling rate needs to be at least above the Nyquist frequency (2x the sampling rate of the highest frequency), and current standards suggest a sample rate 3–4 times the highest frequency component of physiological signal. Advancements in modern computing allow for research-grade equipment to sample far above Nyquist for most of the measures discussed (>2,000 Hz) during data acquisition. Of course, data can always be down-sampled post data collection. As discussed in sections “Heart Activity Quantification” and “Practical Considerations” on heart activity, quantification using wearables can lead to inaccurate assessments (Laborde et al., 2017) due to poor sampling rates, lagged responses, and noisier signals to name a few, which would lead to inaccurate interpretations.

Filters are helpful in getting rid of artifacts and noise not relevant for the physiological signal being processed. For instance, muscle and electrical noise (around 60 Hz) are not meaningful while interpreting EEG and ERP data, and thus data outside the range of interest (typically not higher than 40–50 Hz) can be bandpass filtered. However, if EMG activity, which has a much higher frequency content, is of interest, then bandpass filtering with allow low-pass cutoff at 500 Hz and high-pass cutoff at 20 Hz, is often suitable (van Boxtel, 2001). Visual inspection pre- and post-filtering process can help determine how filtering is affecting a signal. Note that all filters distort the waveform and spectral characteristics, so unnecessary filtering should be avoided and researchers should take care to understand exactly how filters are impacting their data in time and frequency domains.

For each psychophysiological measure discussed, researchers have a growing number of indices that can be examined (for example, for HRV, time-based, frequency-based, and non-linear measures can be derived). Choice of metrics should be carefully evaluated, as some metrics may be more suitable to meet the goals of the study, while others may not be suitable. For instance, some metrics require minimum duration of data and falling short of such requirements will lead to misrepresentative findings (e.g., standard deviation of R-R heart beats or SDRR is considered more accurate when calculated over 24 h vs. 5 min or shorter intervals; Shaffer and Ginsberg, 2017). Such choices should be made a priori, based on the research question of interest and links between a measure and its purported psychological interpretation based on prior research. Such flexibility in multi-modal recording comes at the cost of an increasing number of “experimenter degrees of freedom,” that can lead to inflated Type-I error rates, if a consistent analysis pipeline is not followed. It is also important to use comparable durations of physiological signals across conditions and participants for appropriate interpretation. Finally, great attention to accurate event markers is critical for valid interpretation within and across participants in event-related designs. This can be an issue when using commercial products that are not designed for research purposes.

### Innovation

A limitation of most current psychophysiological research-grade measures is the need for using contact sensors (placed on skin). Non-contact sensors are beginning to be tested in applied settings, which can make physiological data collection even less invasive. For instance, ECG data can be derived from high-quality RGB cameras, or sensors could be placed on the steering wheel and driving seats (but should meet the recommended requirements). While these can potentially be a great approach to counter the limitations of contact sensors, caution is advised while considering them because new limitations or inaccuracies in assessment are possible and further research and testing is required to adopt them in research. Commercial products may not meet the requirements recommended by the scientific community, which can lead to poor data quality and invalid interpretations. For example, smartphone camera-based PPG sensing estimates have poor sampling rate and can lead to inaccurate assessments (Laborde et al., 2017). It is essential to ensure that the guidelines for measures are met before investing time and resources to avoid technical issues in data collection and interpretation. For instance, as discussed earlier, it is critical to collect physiological data with recommended frequency sampling to avoid aliasing (Jennings and Allen, 2016). Only equipment that have been or can be validated against research-grade devices should be adopted for research purposes.

### Classification

Reliable and valid assessment of cognitive states is the groundwork to develop inputs to advance state detection-workload managers and “aware” systems. For instance, a recent study reported a reliable method to elicit stress in naturalistic driving scenarios (Baltodano et al., 2018). Given that one measure may not be enough to reliably measure subtle changes in cognitive state, a multi-method approach is critical to capture state-level variations that may not be apparent through a single measure alone. Research has shown that multi-modal approaches provide a reliable (Schmidt et al., 2011; Borghini et al., 2014; Chen et al., 2017) way to sense and assess cognitive states of motorists in real-world settings. Notably, due to the dynamic nature of the physiological signals, conventional linear approaches are not always appropriate in modeling and predicting cognitive state (Chen et al., 2015). The discussed physiological signals are often non-stationary overall but for the briefest periods of time. As such, innovative methods of combining temporal and spectral resolution (time-frequency analysis) have been developed in some domains (e.g., EEG), but their application to other physiological signals is only in its infancy.

Once data have been processed to remove artifacts or irrelevant noise, machine learning techniques could be trained on these data to identify “risky” sub-optimal levels of cognitive states, such as low-arousal states of drowsiness and fatigue associated with unsafe driving performance. During the training phase, multimodal features extracted from physiological training data could be used to train models to classify observations into high-arousal states (e.g., due to high stress and workload), optimal-arousal state, vs. low-arousal state (e.g., due to drowsiness and fatigue). During the test phase, the fully-specified machine learning algorithm can be tested in terms of its capacity to accurately classify observations into respective arousal states. Indeed, cognitive state detection based on multimodal feature analysis and classifiers have been also used to detect stress (Yang et al., 2016; Chen et al., 2017; Lee et al., 2017b), alertness and drowsiness (Forsman et al., 2013; Correa et al., 2014; Chen et al., 2015; Wang and Chuan, 2016), fatigue (Fu and Wang, 2014; Wang, 2015; Fu et al., 2016; Li et al., 2017; Wang et al., 2017), and workload (Borghini et al., 2014; Yang et al., 2016) in real-time. Such studies have integrated data from more than one measure by conducting multi-modal analysis to extract the relevant features to capture the psychological phenomena at hand. A comparison of multiple classifiers to train & optimize machine learning algorithms can help determine the best fitting model to represent changes in cognitive states that can explain driving performance (Nadeau and Bengio, 2000; Fairclough et al., 2015; Balters and Steinert, 2017; Tran et al., 2017). Thus, utilizing multi-modal physiological signals, models could be trained to learn and predict motorists' sub-optimal cognitive states associated with unsafe-driving behavior.

The optimized machine learning algorithms could accordingly inform advanced state detection managers to trigger warnings or otherwise intervene when sub-optimal cognitive states associated with risky driving behavior are detected (Aidman et al., 2015). The ability to predict unsafe levels of physiological arousal will enable targeted augmentation to modify motorists' cognitive state to promote safer driving behavior (Schmidt and Bullinger, 2017; Schmidt et al., 2017; Aricò et al., 2018). For instance, countermeasures to augment cognitive states, such as thermal stimulation (Schmidt and Bullinger, 2017; Schmidt et al., 2017) and warning signs or verbal communication (Schmidt et al., 2011; Aidman et al., 2015) can be used by an automated system to modify drivers' cognitive state. This may especially benefit vulnerable groups such as inexperienced drivers (Noordzij et al., 2017; Yan et al., 2017) and older (Costa et al., 2017) drivers who may be more susceptible to cognitive overload. Furthermore, a person-centered approach can account for individual differences, such as the role of age, driving profile, trust, and reliance on automation. For instance, a recent study used discriminant analysis to account for motorists' driving-styles and individual difference factors (e.g., gender, age, anxiety, anger) and also identify motorists' EEG and EDA response features to classify motorists' safe vs. risky driving tendencies (Liang and Lin, 2018). This study shows that individual differences can explain variations in driving performance and a customized approach may also help improve model prediction over time by accounting for motorists' characteristics and preferences. For example, the low, normal, and high physiological arousal ranges will vary depending on attributes such as anxious, risky, and distress reduction driving styles of an individual (Liang and Lin, 2018) and prediction of cognitive state-level variations may be more accurate when predictions account for such individual-level variations. Thus, a person-centered approach will improve reliable predictions of cognitive states in real-world contexts by intelligent driving systems.

## Research Applicability in Real-World Settings

As the reviewed literature in section, “Psychophysiological Measures to Assess Cognitive States” suggests, many interrelated states could lead to a similar pattern of findings on a physiological measure (e.g., mental fatigue, drowsiness, lower vigilance, and mind wandering are all sensitive to similar EEG/ERP indices). After considering the overlap across findings from interrelated constructs, in Table 1 we have summarized the expected pattern that each physiological measure will have during a low vs. high arousal state in an applied driving context. There are a few points to consider. First, changes in several related cognitive states can lead to similar changes in arousal. For example, increases in driver workload, stress, or vigilance may occur under different contexts, but may similarly lead to heightened arousal. Second, even though arousal is continuous, we chose to classify driver states into categories of low and high arousal because both extremes are sub-optimal for driving performance. Third, cognitive states are complex and change across time. For instance, in the current review, we have placed mind wandering in a low-arousal state based on similar patterns of findings as drowsiness. However, mind wandering is a convenient short-hand for a more complex constellation of non-externally directed cognitive states (see Smallwood and Schooler, 2006 for a review) and depending on the context, such mind-wandering states can yield states of heightened-arousal as well. Similarly, fatigue can be categorized as high-arousal due to prolonged cognitive overload or it can be passive, because of underload due to monotonous driving conditions, for example (Saxby et al., 2008; Matthews et al., 2019). With further empirical evidence in naturalistic environments, a better characterization of complex cognitive states could be developed.

It is still an open question if interrelated cognitive states could be successfully differentiated from other similar states in naturalistic environments (see Cacioppo et al., 2007 for challenges with psychological inference). However, physiological measures could be used to assess sub-optimal levels of general arousal in real-world settings and intelligent systems can use this information to trigger augmentation strategies even if we cannot fully differentiate between specific cognitive states besides along their arousal axis. We have reviewed how physiological responses across multiple measures can provide a rich array of response data relevant to domains that are of interest to driving researchers (e.g., attention, fatigue, workload, etc.). These measures provide unique information and unique sensitivity to experimental manipulations beyond behavioral responses alone. Thus, their current and future utility in real-world driving research is important. This does not mean that measuring one or even a large number of these measures alone will provide us with a *direct* interpretation of a covert state (e.g., becoming increasingly frustrated about an aggressive driver behind you). Before the state of the research matures to be able to address such a lofty goal as predicting specific cognitive states (Yarkoni and Westfall, 2017), we first need careful on-road experimental work to understand the sensitivity and specificity of these measures to specific changes in driver-relevant states in observational and experimental research in real-world settings. Thus, the focus of the current review is not to claim that measurement of multiple physiological measures in real-world driving could accurately predict motorists' specific cognitive state. Rather, our goal is to summarize the feasibility of each of these measures for integrating high-quality psychophysiological methodology into real-world driving research. Table 1 presents the current working predictions that are expected based on the available literature, but more work is needed to be able to use physiological signals to infer psychological processes. The current review represents a summary of initial steps in that direction.

In Table 2, we have summarized the research applicability of the reviewed psychophysiological measures. Although all of these measures can provide valuable insights in the controlled settings of a lab, some measures are more feasible to use and interpret than others in real-world driving contexts. A few factors that may play a role in determining the practical use of physiological measures in applied settings are: the degree of coupling between the measure and subtle changes in cognitive states, temporal resolution, psychometric reliability, ease of data collection (e.g., setup time), sensitivity to artifacts, and the degree of invasiveness and disruption to normal driving. After considering the available evidence, we have categorized each measure's real-world research applicability into low, medium, or high levels. Moreover, certain measures may be better candidates than others for a near real-time assessment in applied settings. We review the real-world applicability and feasibility of each of the measures in Table 2.

Some promising work suggests that cardiovascular measures may be robust in detecting near real-time changes across multiple domains. Studies have shown that cardiovascular data can reliably detect changes in workload (Mehler et al., 2009, 2012; Lenneman and Backs, 2010; Stuiver et al., 2014), fatigue (Patel et al., 2011; Matthews et al., 2019), and drowsiness (Vicente et al., 2016; Kurosawa et al., 2017). Like any physiological signal, cardiovascular data is susceptible to artifacts that could otherwise lead to inaccurate estimations. However, recent analytical advances have led to an improved use in real-world settings even in the presence of substantial recording artifact. For instance, an analysis approach using short segments of cardiovascular data (e.g., a moving window of 30 s; Stuiver et al., 2012) can be used to detect workload demands during driving (Stuiver et al., 2014). Use of smaller temporal windows of data allow for an investigation of the short-term effects of cognitive state without being overly susceptible to artifacts. Recent work has shown that frequency analysis techniques on ECG data can also be utilized to detect early onset of fatigue (Matthews et al., 2019). While the limitations of PPG discussed earlier still apply, recent preliminary work using near-infrared illumination PPG (which overcomes confounds of illumination and motion-related inaccuracies) while driving seems a promising direction for future practical applications (Nowara et al., 2018). Another recent work has developed a noise-resistant algorithm specifically designed to analyze PPG waveforms (van Gent et al., 2018), which can provide researchers an open-source and validated heart rate analysis software to overcome some existing limitations of PPG data processing, making it more feasible for applied driving research.

EDA has been found to be a robust measure of sympathetic arousal in driving contexts in real-world settings (Mehler et al., 2012; Schneegass et al., 2013; Ruscio et al., 2017). EDA is also easy to set up and collect from a motorist without obstructing the driving process. Even though it has a slower response time and provides only a broad sense of arousal (a combination of workload, stress, fatigue, etc.), EDA in an applied uncontrolled environment can estimate relative changes and periods of stability in sympathetic activity of a motorist with an upper temporal resolution of approximately 3–5 s. For example, recent work found EDA to be suitable in capturing stress-level variations in a real-time unconstrained setting (ElKomy et al., 2017). Feature extraction and pattern recognition algorithms have also shown reasonable success recently in detecting changes in cognitive states (Chen et al., 2017; Liu and Du, 2018). Moreover, adaptive filters have been successfully used to remove motion-related artifacts for automatic and accurate detection (up to 95% sensitivity) of state-level variations in cognition (Affanni et al., 2018). Such recent processing and analytic advances with EDA data has shown its high relevance in applied intelligent automation. For example, a development approach proposed for monitoring driver's fatigue levels and functional state utilizes automated analysis of EDA indices in their detection module to improve intelligent vehicular systems (Liu and Du, 2018; Savchenko and Poddubko, 2018).

EEG, a direct measure of brain's electrical activity, can provide robust measures of cognitive state variations while driving, including levels of drowsiness (Liang et al., 2006; Wei et al., 2018), fatigue (Liu et al., 2015; Fu et al., 2016; Hung et al., 2017), and workload (Dasari et al., 2017; Zander et al., 2017). EEG has high temporal resolution and is a direct measure of brain activity. However, data collection (e.g., longer setup time) and processing in real-world setting (e.g., movement artifacts) can be quite challenging to implement into a real-world driving research protocol (Popescu et al., 2008). At the same time, there have been innovative technological and analytical developments in EEG acquisition. For instance, efforts in brain computer interface applications have utilized a single electrode to classify relaxed vs. cognitive workload phases (Shirazi et al., 2014) and monitor fatigue levels (Morales et al., 2017). Recent work extracted features from a 6-channel EEG dataset to classify mental tasks with up to 83% accuracy rate (Neshov et al., 2018). Other recent work has reported detection algorithms that can be used to accurately classify fatigue (Li et al., 2017; Gao et al., 2018). In other work, a novel approach to detect drowsiness has been proposed which reduces calibration time for a new user by 90% using a hierarchical clustering method, which accounts for inter- and intra-subject variability (Wei et al., 2018). Automatic drowsiness detection algorithms based on only a single target channel can allow real-time neural assessments of cognitive states (Belakhdar et al., 2018). With increasing advancements in sensor development and data processing, we hold an optimistic view of adopting EEG-based measures in driving research, albeit after considerable validation (Kosiachenko and Si, 2017; Krol et al., 2017; Zander et al., 2017; Byrom et al., 2018). Recent work has also shown the applicability of specific ERP components (such as the P300), some of which show good psychometric properties (e.g., Cassidy et al., 2012), and can be adopted to brain-computer interfaces (Piña-Ramírez et al., 2018). Future work and reliable replication of studies are required to ensure EEG and ERPs could be assimilated in human-machine automation interface.

Traditional fNIRS has lower temporal resolution and may additionally be difficult to collect in applied settings. However, recently, mobile-friendly systems have been developed and used in applied domains (von Lühmann et al., 2015) including exercise physiology (Byun et al., 2014), clinical monitoring (Kassab et al., 2018), and infant developmental research (Quaresima et al., 2012). Importantly, these advancements mean that fNIRS measurements can be performed in naturalistic environments without considerable restraint. As the development of ultra-portable systems grows (e.g., battery powered mobile systems, McKendrick et al., 2016), fNIRS will likely form a novel complement to the many other physiological measures discussed here, in part because of its unique capability to image neural hemodynamics and reveal changes in brain activity with improved spatial resolution compared to other portable and non-invasive neurophysiological methods (e.g., EEG; Ahn and Jun, 2017). For instance, a recent study adopted a wearable fNIRS system (with sensors placed on a baseball cap making it less intrusive) to measure cognitive distraction while driving (Le et al., 2018). Thus, while these methods are still in their infancy compared to many of the other methods discussed here, the ability to reveal neural mechanisms of cognitive states in real-world domains such as driving is promising.

Similar to fNIRS, thermal imaging also shows some early promise. It is a non-contact technology that has high relevance in applied settings, including driving (Lee et al., 2018). For example, recent work has shown the validity of thermal imaging in indexing cognitive load. In these studies, changes in nasal and forehead temperatures were observed as a function of task difficulty in a non-driving context (Abdelrahman et al., 2017; Marinescu et al., 2018). However, research in real-world settings is currently limited. Existing preliminary work has focused primarily on understanding the sensitivity of this measure in well-controlled environments. Future work will help qualify the utility and validity of thermal imaging in real-world conditions.

On the other hand, several measures, despite clear utility in a lab environment, may be currently of less use in real-world settings. For example, pupillometry in well-controlled lab settings can provide helpful information in interpreting user state (e.g., Pfleging et al., 2016; Cegovnik et al., 2018). Moreover, with the development of desktop-mounted eye trackers, pupil dilation and constriction can be measured non-invasively and remotely with high spatial and temporal resolution. In lab settings, where features such as luminance can be controlled and measured, recent work has shown success in using pupillometry to examine mental workload in an unconstrained setting (e.g., Lego construction; Bækgaard et al., 2019). In driving, some researchers have suggested that pupil-based measurements are highly relevant for assessment of drowsiness (Maccora et al., 2018). However, detection of pupil diameter in real-world settings with rapidly changing and uncontrollable variations in luminance is a critical confounding factor in the utility of pupillometry in driving (Kassem et al., 2017).

Similarly, EMG can be utilized in lab settings to understand psychological processes. For example, EMG in combination with other psychophysiological measures was recently utilized in detecting fatigue in drivers (Fu et al., 2016; Ma et al., 2018). Preliminary research has also proposed the use of EMG to detect drowsiness (Artanto et al., 2017) and real-time monitoring of muscle activity (Mazzetta et al., 2018). However, in applied settings such as driving, EMG may have only low utility, in part because the necessary motor activity needed to engage in the task (e.g., turning the steering wheel and actuation of break) can cause uncontrolled changes in muscle activity that can be confounded with the psychological variance in EMG, which is an order of magnitude smaller than these artifacts.

At the same time, ongoing methodological developments are resulting in more efficient systems, improved signal-to-noise ratio, and improved signal-processing methods, all of which culminate in rapidly improving the reliability and validity of acquisition across these multiple methodologies. Some attempts to assess cognitive states using multiple methods have been integrated in non-driving domains (ElKomy et al., 2017; Ko et al., 2017; Moghaddam and Lowe, 2019) and multi-method work in real-world driving contexts are already underway (Fu et al., 2016; Brouwer et al., 2017; Zander et al., 2017; Aricò et al., 2018; Belakhdar et al., 2018; Haouij et al., 2018; Paredes et al., 2018; Rastgoo et al., 2018).

Taken together, we have reviewed a growing body of empirical evidence suggesting that physiological measures can be used to sense and assess changes in the cognitive states of motorists during real-world driving. Through this selective review, we believe that the strengths and limitations of adopting physiological measures in driving can clearly extend to other domains such as the use of aircraft, trains, and ships. Furthermore, we see growing promise for the application of covert monitoring methods like those reviewed above with the increasing rise in semi-automated technology, where motorists will become less directly involved in the driving process. As such, the development of intelligent driving assistance systems will need to utilize non-behavior-based measures to index covert cognitive states of a motorist in the absence of any overt behavior. The physiological measures reviewed above have the potential to detect sub-optimal arousal levels associated with risky driving behavior and inform state detection-workload managers and “aware” systems to trigger warnings or intervene, resulting in a closed-loop system in the absence of any overt-driving behaviors. Before we reach such a future however, the field needs to adopt rigorous standards for the use of psychophysiological measurement in real-world settings. We hope to see a future of increased collaboration and integration of basic psychophysiology, human factors, and traffic safety research. Such integration is necessary to advance the development of effective human-machine driving interfaces and driver support systems, with the ultimate goal of improving traffic safety.

## Author Contributions

All authors listed have made a substantial, direct and intellectual contribution to the work, and approved it for publication.

### Conflict of Interest Statement

The authors declare that the research was conducted in the absence of any commercial or financial relationships that could be construed as a potential conflict of interest.
